# Anesthesia mumps with airway obstruction after radical nephrectomy: A case report and literature review

**DOI:** 10.3389/fsurg.2022.1039362

**Published:** 2023-01-06

**Authors:** Wanqiang Li, Zhengquan Liao, Ling Yao, Lusheng Zhang, Xuesong Li, Ziqiang Dong

**Affiliations:** ^1^ Department of Urology, the First College of Clinical Medical Science, China Three Gorges University, Yichang Central People's Hospital, Yichang, China; ^2^ Department of Urology, Yidu People's Hospital, Yichang, China; ^3^ Department of Intensive Care Unit, the First College of Clinical Medical Science, China Three Gorges University, Yichang Central People's Hospital, Yichang, China; ^4^ Department of Urology, Peking University First Hospital, Institute of Urology, Peking University, National Urological Cancer Center, Beijing, China

**Keywords:** postoperative care, radical nephrectomy, sialadenitis, anesthesia mumps, airway obstruction

## Abstract

Anesthesia mumps have rarely been reported. This article presents the diagnosis and treatment of a case of anesthesia mumps with airway obstruction in the urology department and reviews previous cases of the disease. A 58-year-old man had a history of hypertension and diabetes, and his blood pressure and glucose levels were well controlled. He underwent laparoscopic radical nephrectomy for a right renal tumor. Postoperatively, a swelling of approximately 5 × 4 cm was observed in the left parotid region and left eyelid, no palpable crepitation was detected, and the skin overlying the left parotid gland was mildly hyperemic and tender. Enhanced computed tomography of the head and neck revealed obvious swelling of the laryngopharyngeal airway, and electronic laryngoscopy showed narrow airway. Laboratory test results including white blood cell count, C-reactive protein, serum amylase, and lipase levels were normal. Glycosylated hemoglobin level was 6.8%, and the salivary culture from Stensen's duct was negative. The patient was managed with endotracheal intubation and a ventilator to maintain breathing along with anti-infection, expectorant, and symptomatic treatment. The swelling in the left parotid gland gradually resolved without recurrence, and the patient was extubated on the 7th postoperative day. In this case, the pathophysiology of anesthesia mumps may have been related to the incorrect positioning of the thick short neck and the use of a head ring, which can result in the squeezing of vessels. In most cases, the salivary gland swelling resolves with observation and symptomatic treatment. In patients with anesthesia mumps, emergency airway management and careful observation are necessary if upper airway obstruction occurs. This case report should increase awareness of anesthesia mumps and its complications among anesthesiologists, surgeons, and postoperative caregivers.

## Introduction

Transient inflammation and swelling of the salivary glands is a rare complication after general or epidural anesthesia and is defined as anesthesia mumps (1). It is usually benign and can develop during surgery or, more commonly, a few hours postoperatively, and usually resolves without sequelae within few days. In rare cases, it can lead to severe symptoms that require emergency intervention (2). Anesthesia mumps has been reported after various surgical procedures (2–22) (Table 1). Herein, we report a case of anesthesia mumps, which involved the left parotid gland and caused severe facial edema and airway stenosis, after laparoscopic radical nephrectomy in the urology department.

**Table 1:** A review of anesthesia mumps cases reported till date.

Sex	Age (years)	Department	Symptoms	Treatment	Prevention methods	Reference
Male	59	Vascular surgery	Bilateral parotid swelling, airway obstruction	Tracheal intubation	Postoperative follow-up observation	Hamaguchi et al. (2018) (2)
Female	36	Thyroid surgery	Bilateral parotid swelling	No treatment	Paying attention to patient position, endotracheal tube management, and hydration	Cozzaglio et al. (2017) (3)
Male	6	Endoscopy	Left parotid swelling with mild tenderness	No treatment	Using adaptive-shaped soft pads, changing the head and neck position	Jan et al. (2020) (4)
Male	8	Otolaryngology	Painless swelling on the right parotid gland	Conservative treatment	Postoperative follow-up observation	Bayir et al. (2015) (5)
Female	46	Gynecology	Painless swelling on the right parotid gland	Conservative treatment	Postoperative follow-up observation	Özyurt et al. (2019) (6)
Female	40	Gynecology	Left parotid swelling	Conservative treatment	A gentle procedure is required in usual face mask handling	Kwon et al. (2015) (7)
Male	60	ICU	Left parotid swelling	Conservative treatment	Frequent head and neck position checking	Ghatak et al. (2013) (8)
Female	54	Urology	Left parotid swelling	Conservative treatment	Early diagnosis and proper management	Erkiliç et al. (2017) (9)
Male	40	Psychiatric	Bilateral parotid swelling	Warm compress	Postoperative follow-up observation	Katz et al. (2017) (10)
Male	52	Spine surgery	Left parotid swelling	Conservative treatment	Proper use of the anesthetic medication and technique	Asghar et al. (2015) (11)
Male	33	Psychiatric	Bilateral parotid swelling	Nonsteroidal anti-inflammatory drug	Adequate hydration	Akçaboy et al. (2011) (12)
Female	16	Neurosurgery	Right parotid swelling, airway narrow	Transnasal fiberoptic intubation, steroids, and antibiotics	Careful attention to the patient's history, early diagnosis	Quinn et al. (2012) (13)
Female	57	Plastic surgery	Left parotid swelling	Symptomatic therapy	Paying attention to medical history and physical examination	Tekelioglu et al. (2012) (14)
Female	3	Otolaryngology	Left parotid swelling	Symptomatic therapy	Observation, hydration, and antibiotics	Özdek et al. (2014) (15)
Male	26	Orthopedics	Left parotid swelling	No treatment	Using soft pads and keeping the patient normovolemic	Baykal et al. (2009) (16)
Male	55	Thoracic surgery	Left parotid swelling	No treatment	Adequate hydration	Serin et al. (2007) (17)
Male	80	Endoscopy	Left parotid swelling	Hydration and antibiotics	Observation and antibiotics	Bahadur et al. (2006) (18)
Female	45	Orthopedics	Right parotid swelling	Symptomatic therapy	Minimal turning of the neck and proper padding	Narang et al. (2010) (19)
Female	73	Neurosurgery	Left parotid swelling	Tracheal intubation	Maintain a safe artificial airway	Cavaliere et al. (2009) (20)
Female	35	Urology	Left parotid swelling	Symptomatic therapy	Using adaptive-shaped soft pads, changing the head and neck position	Postaci et al. (2012) (21)
Male	52	Orthopedics	Right parotid swelling	Symptomatic therapy	Using adaptive-shaped soft pads, changing the head and neck position	Liu et al. (2007) (22)
Female	53	Orthopedics	Right parotid swelling	Symptomatic therapy	Proper use of anesthetic medication and technique	Liu et al. (2007) (22)

ICU, intensive care unit.

## Case report

A 58-year-old man underwent laparoscopic radical nephrectomy for a right renal tumor in the urology department. The patient had a history of hypertension for 3 years and type 2 diabetes for 17 years, and his blood pressure and glucose levels were well controlled. The patient's body mass index (BMI) was 32.1.

In the 2nd day after admission, the patient underwent laparoscopic radical right nephrectomy under general anesthesia. Anesthesia was induced using propofol (2 mg/kg) and fentanyl (1 µg/kg). Endotracheal intubation (internal diameter 8 mm) was performed after muscle relaxation with vecuronium (0.1 mg/kg), and sevoflurane (1MAC) was used for anesthesia maintenance. The patient was placed in the left lateral position with his head on a soft gel pad, and with the waist as the fulcrum, the patient's lower limbs and head were lowered by 20° each. The surgical time was 5 h and 47 min.

**Figure 1:**
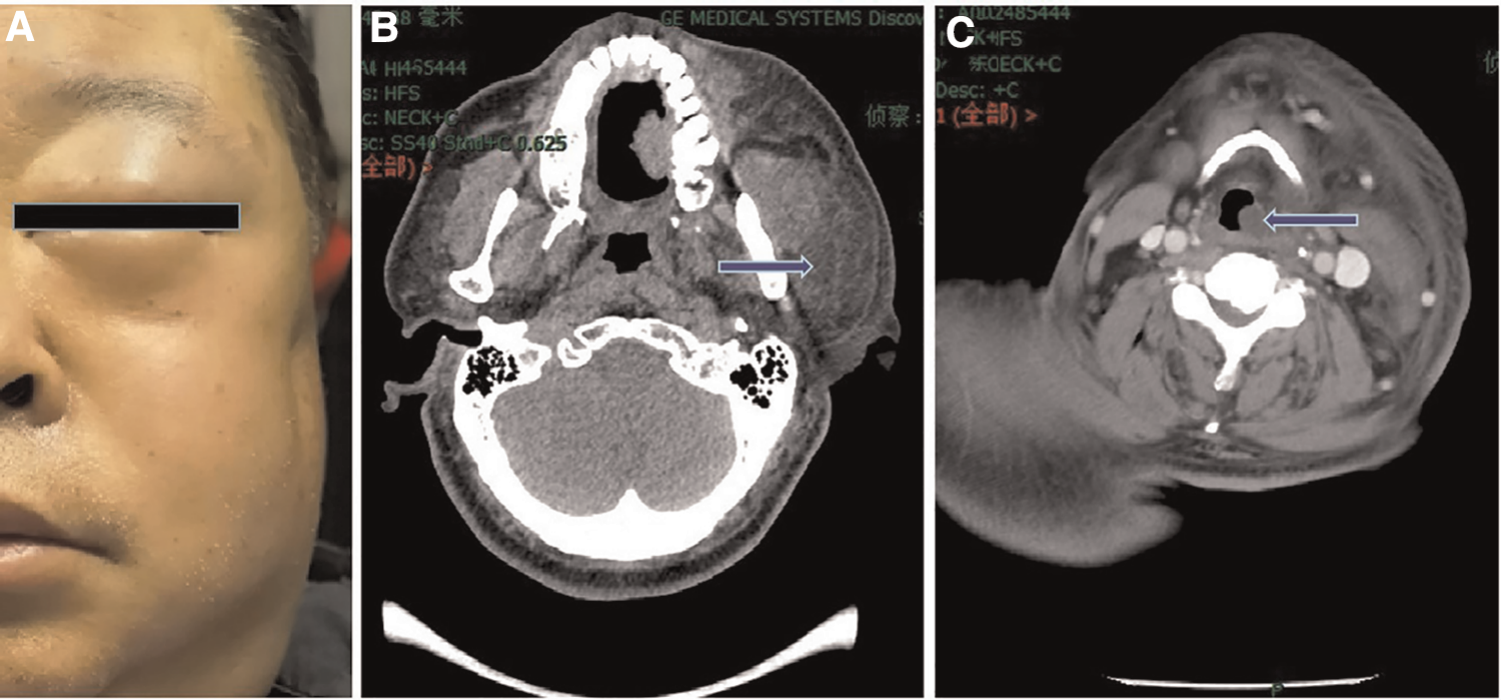
Clinical appearance and imaging findings of the patient. (**A**) Photograph of the patient showing swelling of the left eyelid and parotid gland area. (**B**) Enhanced CT image showing significant enlargement of the left submandibular soft tissue and parotid gland (arrow). (**C**) Enhanced CT image showing the involvement of the left parapharyngeal space and airway stenosis (arrow).

Shortly after the patient was shifted to the anesthesia recovery room, a swelling of approximately 5 × 4 cm was observed in the left parotid region, while the body temperature remained normal. The facial swelling was further aggravated at 3 h postoperatively; swelling was found on the left eyelid and forehead (Figure 1A), and the overlying skin of the left parotid gland area was mildly hyperemic and tender. However, no crepitation on palpation was detected. Enhanced computed tomography (CT) of the head and neck showed obvious swelling of the soft tissue from the left periorbital area to the left submandibular region. The swelling involved the left parapharyngeal space, uvula, oropharynx, epiglottis, and laryngopharyngeal airway (Figures 1B,C). Electronic laryngoscopy showed edema in the left lateral pharyngeal wall, epiglottis, and supraglottic tissue, and the glottis was partially obscured. The patient developed shortness of breath 5 h postoperatively. Considering upper airway obstruction, emergency endotracheal intubation was performed, and the patient was transferred to the intensive care unit for monitoring and treatment.

Laboratory examination revealed a normal white blood cell count; C-reactive protein, serum amylase, and lipase levels were normal; glycosylated hemoglobin level was 6.8%; salivary culture of Stensen's duct was negative; and the pathological diagnosis of the tumor specimens was renal cell carcinoma.

The patient was diagnosed as anesthesia mumps and treated with endotracheal intubation and a ventilator to maintain his breathing, along with anti-infection treatment, an expectorant, and blood glucose control. Repeat CT 4 days postoperatively showed resolution of the swelling in left temporal, facial, and cervical soft tissue and parapharyngeal space. Laryngoscopy confirmed the absence of redness or swelling of the nasopharynx, oropharynx, laryngopharynx, or epiglottis, and no airway obstruction was observed. The swelling left parotid gland gradually resolved without recurrence within 7 days postoperatively, and the patient was extubated without incident.

## Discussion

The etiology of parotid gland enlargement is complex and varied (1). Anesthesia mumps is an acute swelling of the unilateral or bilateral parotid glands after surgery (7), which was first described in 1960, and its incidence has been reported to be 0.16%–0.2% (23). The exact pathophysiology of anesthesia mumps is not clear, and many factors are thought to play a role. First, the loss of muscle tone caused by muscle relaxants and positive pressure mask ventilation during anesthesia is considered a factor. During recovery from general anesthesia, the significant increase in positive pressure in the mouth due to nervousness, coughing, and sneezing while the patient is still receiving positive pressure ventilation facilitates the retrograde passage of air through Stenson's ducts into the parotid gland (1). Second, the perioperative use of anticholinergics, phenothiazines, antihistamines, barbiturates, beta-blockers, and preoperative dehydration may be inducing factors. This can lead to obstruction of the salivary ducts by reducing salivary secretion and increasing the viscosity of secretions (19). Third, Mandel and Surattanont believed that activation of the pharyngeal reflex stimulates the parasympathetic nervous system, which in turn leads to vasodilation and hyperemia in the parotid gland (24). Another possible explanation is that the patient's parotid canal becomes blocked due to overrotation of the head and compression of the parotid gland during long periods of surgery (7). In addition, some medical conditions, such as diabetes, hypothyroidism, hepatorenal failure, Sjogren's syndrome, depression, and malnutrition, also increase the risk of anesthesia mumps (25).

In our patient, transient parotid secretion obstruction due to dehydration was not considered a possible cause of swelling as good intraoperative hydration was ensured. Moreover, the patient had no history of parotid or salivary calculi, or neck masses, and excretory duct obstruction was ruled out as CT confirmed the absence of calculi, air, or duct dilation. Bacterial parotitis was excluded due to normal infection indicators, and no purulent discharge was observed from Stenson's duct orifice. Adverse drug reactions were also less likely as the patient's body temperature and eosinophil count were normal, and no skin rash was observed. Furthermore, drugs related to parotid swelling were not administered during anesthesia. Therefore, this condition may have occurred due to the compression of vessels owing to the short, thick neck of the obese patient, which affected perfusion of the area supplied, leading to anesthesia mumps. The involved salivary gland was on the side of the face that was pressed against the operating table, and the swelling may be associated with prolonged surgery. In obese patients, the lateral decubitus position may cause venous congestion due to twisting of veins and/or gland ischemia due to compression of the arteries. Venous congestion can explain immediate facial swelling after surgery, and ischemic salivary glands usually present with unilateral swelling and pain. Depending on the degree of swelling, pain can vary from mild and bearable to unbearable (26). In this case, the use of a head ring and extreme neck extension may be the reasons for the development of anesthesia mumps.

Previous reports have shown that in the majority of cases, anesthesia mumps resolved spontaneously in the short term. In many patients, undetected anesthesia mumps resolved without any treatment, while in some cases, hydration and symptomatic treatment were sufficient to treat mumps (24). However, our patient developed severe airway obstruction, and tracheal intubation was performed. No case of anesthesia mumps with airway stenosis has been previously reported following urological surgery. Emergency tracheostomy may be an option if intubation is difficult (27); however, it is difficult to identify the location of tracheostomy in obese patients with a short neck or neck swelling.

Several measures have been proposed to prevent the occurrence of anesthesia mumps, include reducing or avoiding the use of anticholinergic drugs prior to surgery, smoothing intubation and extubation to avoid mechanical irritation, and maintaining optimal hydration during surgery (8). In the present case, a properly shaped soft pad to avoid direct compression of the parotid gland and ducts, and a tension-free head and neck position to maintain normal blood circulation might have prevented the development of anesthesia mumps.

## Conclusion

In summary, anesthesia mumps is a rare complication following urological surgery. It is usually noticed in the recovery room, and surgeons, anesthesiologists, and postoperative caregivers should be aware of this complication. It is important to pay attention to patients with predisposing conditions, such as obesity and diabetes, placed in the lateral or prone positions during long surgeries. Particular attention should be paid to the endotracheal tube insertion and removal and rehydration therapy. When anesthesia mumps with airway obstruction occurs, it is necessary to inform the patient of the condition and take appropriate treatment measures in a timely manner.

## Data Availability

The original contributions presented in the study are included in the article/Supplementary Material, further inquiries can be directed to the corresponding author.
